# A method for longitudinal, transcranial imaging of blood flow and remodeling of the cerebral vasculature in postnatal mice

**DOI:** 10.14814/phy2.12238

**Published:** 2014-12-18

**Authors:** Annelise Letourneur, Victoria Chen, Gar Waterman, Patrick J. Drew

**Affiliations:** 1 Department of Engineering Science and Mechanics, Center for Neural Engineering, Pennsylvania State University, University Park, Pennsylvania; 2 CNRS, CEA, Université de Caen Basse‐Normandie, UMR 6301 ISTCT, CERVOxy. GIP CYCERON, Caen, France; 3 Department of Neurosurgery, Pennsylvania State University, University Park, Pennsylvania

**Keywords:** Cerebral vasculature, development, imaging, two‐photon

## Abstract

In the weeks following birth, both the brain and the vascular network that supplies it undergo dramatic alteration. While studies of the postnatal evolution of the pial vasculature and blood flow through its vessels have been previously done histologically or acutely, here we describe a neonatal reinforced thin‐skull preparation for longitudinally imaging the development of the pial vasculature in mice using two‐photon laser scanning microscopy. Starting with mice as young as postnatal day 2 (P2), we are able to chronically image cortical areas >1 mm^2^, repeatedly for several consecutive days, allowing us to observe the remodeling of the pial arterial and venous networks. We used this method to measure blood velocity in individual vessels over multiple days, and show that blood flow through individual pial venules was correlated with subsequent diameter changes. This preparation allows the longitudinal imaging of the developing mammalian cerebral vascular network and its physiology.

## Abstract (precis)

We developed a technique to longitudinally image blood vessels in the neonatal mouse cortex transcranially using two‐photon microscopy. The blood vessels on the surface of the brain undergo substantial pruning after birth. Blood flow through a vessel was correlated with the subsequent diameter change of the vessel.

## Introduction

During development and growth, the vascular system of vertebrates must change to accommodate the metabolic needs of the organism. The structure of the vasculature is not genetically hardwired (Jones et al. 2006), and it is thought that the mechanical forces exerted by flowing blood guide vascular remodeling (Culver and Dickinson 2010). Understanding the relationship between mechanical forces and subsequent vascular change requires longitudinal imaging at the single vessel level. At early stages of embryonic development, where longitudinal imaging is possible in fish and chicks over the time‐scale that remodeling takes place, there is good evidence that the fluid forces play an important role in correct assembly of the heart and blood vessels (Hove et al. 2003; Lucitti et al. 2007; Buschmann et al. 2010; Chen et al. 2012; Udan et al. 2013). As the development of the vasculature in mammals, particularly in the brain, continues into adolescence (Craigie 1925), it is important to understand if hemodynamic forces play a similar role in shaping postnatal vascular changes.

In the mouse brain, the first few postnatal weeks are a time of drastic changes in neural responsiveness (Borgdorff et al. 2007; Smith and Trachtenberg 2007; Golshani et al. 2009), morphology (Portera‐Cailliau et al. 2005), and plasticity (Woolsey and Wann 1976; Hensch 2005; Erzurumlu and Gaspar 2012). In parallel to these profound changes in neuronal connectivity and organization, the vascular system of the brain undergoes extensive remodeling (Craigie 1925; Wang et al. 1992). During this time, there is an increase in capillary turnover within the parenchyma (Harb et al. 2013), which is thought to be controlled by neural activity (Whiteus et al., 2014). Additionally, the many arterial‐venous shunts in the pial vasculature are pruned (Murphy et al. 2008, 2012). Particularly noticeable is the change in the pial venous system of the brain, which in mice starts as a dense plexus at birth, and undergoes a removal of a majority of the surface veins over the next few weeks (Wang et al. 1992).

Here, we describe a neonatal reinforced thin‐skull window, a modification of the polished and reinforced thin‐skull (PoRTS) window (Drew et al. 2010b; Shih et al. 2012a,b), for longitudinally imaging large areas (>1 mm^2^) of the developing cerebral vasculature for up to 5 days. Thin‐skull imaging methods (Christie et al. 2001; Grutzendler et al. 2002) do not cause inflammation (Xu et al. 2007), but only allow imaging of a small area, and can require invasive rethinning of bone (Yang et al. 2010). Imaging over several hundred square microns is necessary to capture the changes in the structure of the extended pial vasculature (Blinder et al. 2010, 2013). While larger windows can be made with craniotomies, craniotomies cause inflammation (Xu et al. 2007; Drew et al. 2010a,b; Cole et al. 2011; Lagraoui et al. 2012), which will disrupt normal vascular development. Craniotomies increase pulsation‐related movement in the brain (Paukert and Bergles 2012), and have long been known to drive angiogenesis in the adult brain (Sohler et al. 1941; Arieli et al. 2002; Drew et al. 2010b). Skull removal rapidly changes the mechanical properties of brain tissue (Hatashita and Hoff 1987), which are important for proper neural development (Franze et al. 2012). Lastly, there is evidence that craniotomies cause cortical spreading depression (Brennan et al. 2007; Chang et al. 2010), making it important to leave the skull intact to insure normal neural and vascular development during the imaging period. Using our neonatal reinforced thin‐skull window technique, we are able to longitudinally image vascular structural and physiological changes over a large area. We observed in awake mice that red blood cell velocities in the pial vascular plexus increases with age, and using longitudinal imaging, we determined that the mechanical forces a blood vessel experience were weakly correlated with their subsequent change in diameter.

## Methods

### Animals and surgery

All procedures were approved by the Penn State Institutional University Animal Care and Use Committee. Mice were housed under 12:12 h light/dark cycle with food and water ad libitum. Breeders were maintained on a high fat diet.

Both male and females of three different mouse strains, Swiss Webster, C57BL/6, Cx3cr1‐GFP (Jung et al. 2000; Davalos et al. 2005; Nimmerjahn et al. 2005) were used. All mice were obtained from Jackson Labs, Charles River, or bred in our animal facility. Both Swiss Webster and C57BL/6 were used for imaging experiments, and data were analyzed together as no major differences were observed between the two strains. To acclimate the dam to the smells associated with handling, paper pieces impregnated with the smell of the products used during experiments were placed in the cage several days prior to surgery. Dams were acclimated to the surgery room for a few hours a day for several days before the surgery. Great care was taken not to touch the pups without clean gloves, and to disinfect the surfaces the pup may contact during the procedures. When surgery was performed on one pup, all pups from the litter were removed from the cage and placed in an incubator (Brinsea Products Inc, TLC‐40 Advance Parrot Brooder) that was kept at 35°C and 25% humidity. Immediately following the procedure, the pup that underwent surgery was allowed to recover in the incubator until it was fully mobile. The pup was then gently rubbed with the bedding from the home cage and returned to the home cage with the rest of the litter. This reduced the rate of rejection by the dam.

For surgery, anesthesia was induced with 5% isoflurane in oxygen, and maintained at 1–3%. The pup was placed on a tissue on top of a homeothermic heating blanket (507220F Harvard Apparatus), and covered by a moistened tissue to avoid drying the skin. The rectal temperature was monitored using an extra small probe (IT‐23 Flexible microprobe type T 3′, Physitemp Instruments Inc. + TH‐5/AOP Thermalert Monitoring Thermometer with Analog Output, Physitemp), and core temperature was maintained at 36–37°C. Additional warming was supplied by a heating lamp (Harvard apparatus, 727562 Heat Lamp HL‐1). The head was immobilized with a head‐shaped mold of modeling clay.

After cleaning the scalp with betadine, an incision was made down the midline of the scalp, followed by a second incision perpendicular to the midline on the hemisphere to be imaged. After gently retracting the scalp, a 0.50 mm burr (Fine Science Tools) was used to remove the periosteum from the surface of the skull. We targeted the windows over the visual cortex, 1–2.5 mm lateral and 3–6 mm posterior to Bregma, depending on the age of the pup (Paxinos et al., 2006). The skull was thinned at the window site using 0.50 mm burrs and a Foredom Drill (Dental Micro Motor Drill with foot‐pedal control, Foredom). During this thinning, the skull was constantly kept moist with cold saline. The burr was changed frequently (2–4 times per animal) to prevent residue from sticking and to avoid dulling the bit. Great care was taken to avoid applying too much pressure, which can induce bleeding, and to only thin for a few seconds at a time to prevent heating. While thinning the skull, the burr was kept parallel to the skull, and the bit was moved with the direction of burr rotation to minimize chatter. The skull was thinned to a uniform thickness over an area slightly larger than the final window area, so that the cover slip could be placed flush with the skull surface. The skull is not completely formed at the age of window implantation, making it permeable to the solvents used in most acrylic cements, so we avoided them altogether.

**Figure 1.**
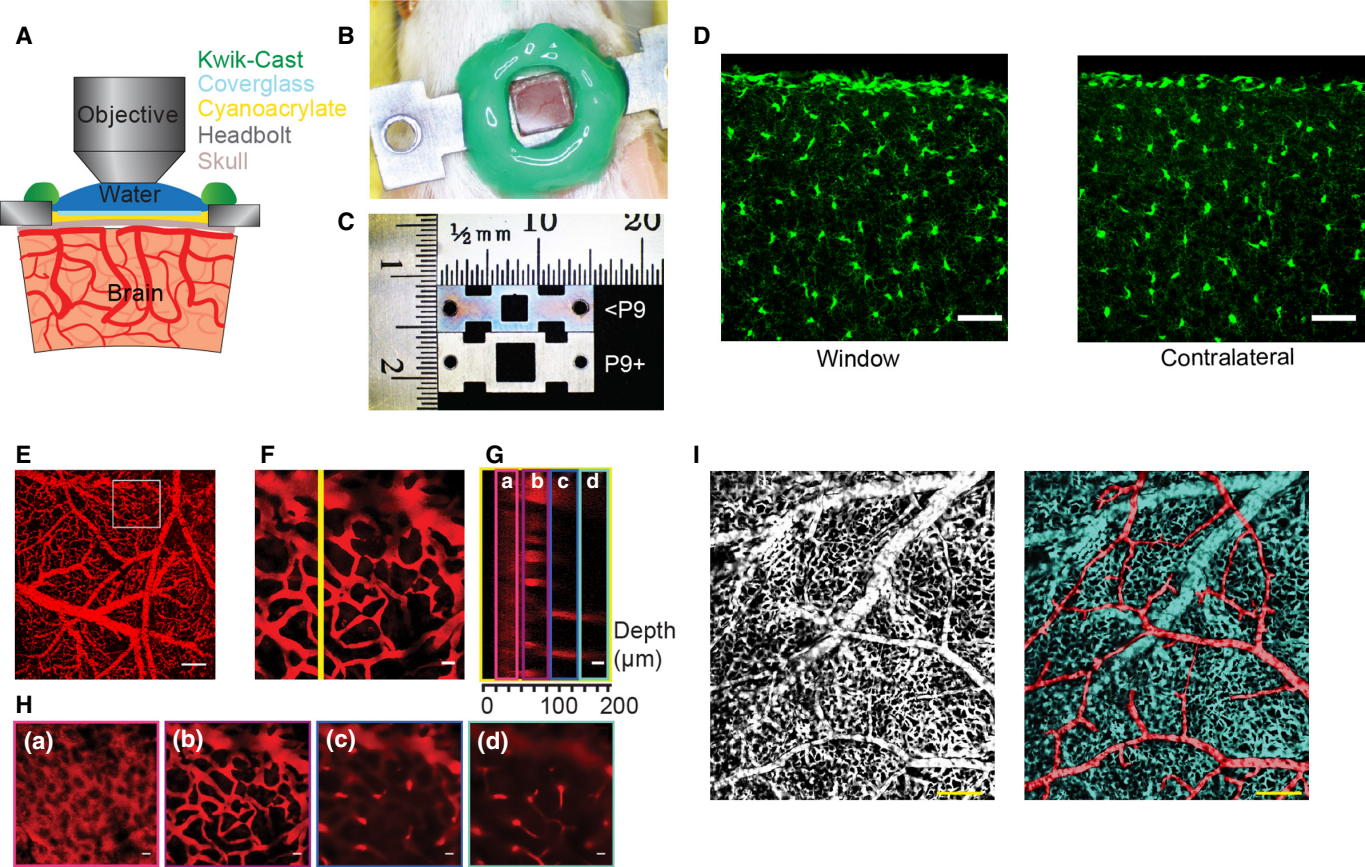
Neonatal reinforced thin‐skull window and histology. (A) Schematic of the thinned and reinforced skull window for imaging the neonatal brain. (B) Photograph of the implanted window and headbolt in a neonatal mouse. (C) Image shows a photograph of the two types of headbolts used. The upper headbolt was used for mice younger than P9, the lower headbolt was implanted in P9 or older mice. The larger skull in older mice enables a larger window to be made. (D) Histological images of microglia. A window was implanted in a 7 day‐old Cx3cr1‐GFP mouse and assayed 4 days later. The left shows a section taken from the cortex under the window, the right is from the contralateral hemisphere. The window implantation did not induce an inflammatory response, as measured by changes in microglia morphology in the cortex. Scale bar is 50 *μ*m. (E) Image of showing maximum projection of a stack of FITC‐dextran filled vasculature taken with a 4× objective in a P7 mouse. Scale bar is 200 *μ*m. (F) Maximum projection of a stack taken in the area denoted by the square in (E). (G) Y‐Z slice through the yellow line in (F). Lowercase letters and colored boxes denote Z‐stack sections along which maximum projections have been made, which are shown in (H). (H) Maximum projections through thin Z sections showing: skull autofluorescence (a), pial vessels (b), and intracortical vessels and capillaries (c and d). The skull was approximately 25 *μ*m thick. Scale bars in F–H, 20 *μ*m. (I) Left, window in a P3 mouse showing the extensive venous plexus present at this age. Right, the same image has been false colored to show arterioles (red) and venous plexus (blue). Scale bars 200 *μ*m.

Once the skull was thinned to approximately 25 *μ*m (Fig. 1G), the window area was cleaned with saline and allowed to dry completely. A drop of cyanoacrylate was applied to the window area, carefully avoiding the creation of bubbles, and a precut piece of glass was gently laid down over the thinned region. Care was taken to lay down the window at the desired place and orientation as it cannot be moved once in contact with the glue. A small amount of pressure was applied to the glass to attach it to the skull. The periosteum was gently removed from the spot where the headbolt was attached with cyanoacrylate. Depending on the age of the pup, different headbolts were used (Fig. 1C). Once the headbolt was firmly affixed, the skin was closed using surgical glue (Vetbond Tissue Adhesive). To protect the window from being damaged by the mother and to hold the meniscus during imaging (Fig. 1A), a well was built around the window with a low viscosity silicone elastomer (Kwik‐Cast Sealant, 2 part, WPI), which can be easily removed or replaced as needed.

The main differences between the reinforced thin‐skull windows for neonates described here and the PoRTS window in adults were the omission of the polishing step and the use of silicone elastomer rather than cement. In preliminary experiments, we found that the polishing step often induced hematomas in neonates. In subsequent animals we skipped the polishing step, which reduced the maximal imaging depth, but did not impede the imaging of the surface vasculature.

Using the above procedures, we used a total of 93 neonates, both C57 and Swiss Webster. Of these, 67 (72%) of the window implantations and subsequent imaging were successful. In 10 (11%) animals various technical factors (loss of headbolt, dye injection failure, etc.) prevented imaging. A further 16 (17%) pups were either killed by the dam or had the window damaged by the mother's grooming. Among successful experiments, 22 were imaged for 1 day, 15 were imaged for 2 days, 8 for 3 days, 14 for 4 days and 8 for 5 or more days. Thus, 48% of the mice in which we implanted windows could be successfully imaged for 2 or more days.

### Imaging procedure

Imaging was performed using a two‐photon laser scanning microscope (Sutter MOM and Mai‐Tai, HP Spectra‐Physics), controlled by MPScan software (Nguyen et al. 2006). The laser was tuned to 800 nm or 900 nm depending on the dye used (fluorescein or Texas‐red) during the experiment. The laser power exciting the sample through the objective was varied between 10 mW and 150 mW depending on imaging depth. Depending on the field of view, we used a 4× 0.15 NA air objective, or 10× 0.3 NA, 20× 0.5 NA or 40×, 0.8 NA dipping objectives (Olympus, Center Valley, PA).

Prior to imaging, the mouse was briefly anesthetized with isoflurane and retro‐orbitally injected with 0.015 mL of a solution of 5% (wt/vol) 70 kDa or 150 kDa fluorescein‐conjugated dextran (Sigma, St. Louis, MO) or 10% Texas Red‐Dextran in filtered sterile saline that had been warmed to body temperature. The retro‐orbital injections in the neonatal mouse were performed using a 0.3 mL insulin syringe with 33G needle.

The animal was then imaged under the two‐photon microscope while awake, in order to avoid the disruptive effects of anesthesia on the cardiovascular system (Drew et al. 2011; Masamoto and Kanno 2012). During imaging, the animal's body temperature was monitored and an infrared heating lamp was used to maintain a normal temperature. Tiled image stacks with 1–5 *μ*m spacing along the *z*‐axis were taken for anatomical data, linescans along vessel axis were taken to determine red blood cell velocity (Drew et al. 2010a; Shih et al. 2012a). Linescans were made for 50–150 sec to insure that at least 40 sec of representative flow, without movement artifacts, was obtained from each vessel. Vessels were chosen for scanning based on their proximity to anatomically distinct structures so that they could be located in subsequent imaging sessions. We purposefully selected vessels of a wide variety of diameters and baseline flow speeds for velocity measurements. After imaging, the pup was rehydrated with an i.p. saline injection (0.01 mL) and placed in the incubator for 15 min before being returned to the home cage.

### Data analysis

>For anatomical measurements, the maximum projection of z‐stacks were made (ImageJ, National Institutes of Health) and tiled. Red blood cell velocities were extracted from linescans using the Radon transform method (Drew et al. 2010a), or for faster flowing vessels, a correlation‐based algorithm (Kim et al. 2012). Vessel diameter were measured using custom‐written Matlab code (Drew et al. 2011) or the ImageJ DIAMETER plugin (Fischer et al., 2010).

### Histology

At the conclusion of experiments, mice were deeply anesthetized with isoflurane and intracardiacly perfused using a peristaltic pump. A perfusion canula adapted for neonatal mice (24 G needle for mouse's pups from P1 to P5, 22 G needle from P7 to P15) was used to perfuse the heparinized saline solution (10 UI heparine/1 mL saline) and the 4% paraformaldeyde in 0.1 mol/L phosphate buffer, pH 7.4. The brain was carefully removed and placed in PFA for 24 h before being sunk in 30% sucrose. Brains sections (30 *μ*m) were then cut using a freezing microtome.

### Statistics

Vessel diameter change‐shear rate relationships were fit in Matlab (Mathworks, Natick, MA).

## Results

### Large‐scale, longitudinal imaging of the pial vasculature

We developed a methodology to longitudinally, transcranially image the cortical vasculature in very young (P2 and older) neonatal mice, using a reinforced thinned‐skull window. A schematic of the imaging window in coronal cross‐section is shown in Fig. 1A. The thinned skull was protected with a glass coverslip attached with cyanoacrylate, and by a silicone well (Fig. 1A and B). The headbolt was attached to the skull using cyanoacrylate glue. Unlike in the adult polished and reinforced thin‐skull (PoRTS) windows (Drew et al. 2010b; Shih et al. 2012b; Knowland et al. 2014), dental acrylic was not used to build a well to hold the water meniscus for the dipping objective, but a fast‐setting, bio‐inert silicone was employed instead. The silicone well has the additional function of protecting the window from mechanical trauma that might be caused by the mother or other pups stepping on the head of the implanted neonate while it is in the home cage. As the size of the mouse increases greatly in the first few postnatal weeks (Eisen 1976), a slightly larger titanium headbolt with a bigger window area was used for older mice (Fig. 1C). The thinned‐skull window implantation in neonates does not cause activation of microglia within the parenchyma (Fig. 1D), as seen with chronic cranial windows where the skull is removed (Xu et al. 2007).

Using these neonatal thinned‐skull windows, we were able to image the capillary bed within the cortex, >100 *μ*m deep in the brain (Fig 1E–H). This example mouse (age P7) displayed the vascular pattern typical of the neonatal cortex. There are many pial veins interconnected on the surface, clearly visible in the maximum projection of a higher magnification Z stack (Fig. 1F). In a Y‐Z view (the plane is denoted by the yellow line in Fig. 1F), the autofluorescence from the skull was visible above the pial vessels, as well as the deeper vessels below them. The skull of the neonate has a mottled pattern (Fig. 1Ha), characteristic of the mineralization pattern of developing cranial bones (Percival and Richtsmeier 2013). Below the skull, the pial vasculature (Fig. 1Hb) and capillaries (Fig. 1Hc and Hd) are visible. Typically, capillaries were visible down to ~200 *μ*m below the pial in the thin skull window, somewhat less than in an adult (Drew et al. 2010b). Some of this reduced imaging depth relative to PoRTS windows in adults was likely due to increased number of blood vessels at the surface, as hemoglobin absorbs at both the excitation and emission wavelengths used, casting “shadows” that reduce the fluorescence signal from the cortex below surface blood vessels (Haiss et al. 2009; Shen et al. 2012), as well as the lack of polishing. In the neonate, as the entire cortical surface was covered by small, shadow‐casting vessels, which tends to counteract the reduced light scattering in juvenile brain tissue (Oheim et al. 2001). We were able to generate large windows, even in very young mice. An example from a P3 mouse is shown in Figure 1I. In general, the size of the window was only limited by the curvature of the skull.

**Figure 2.**
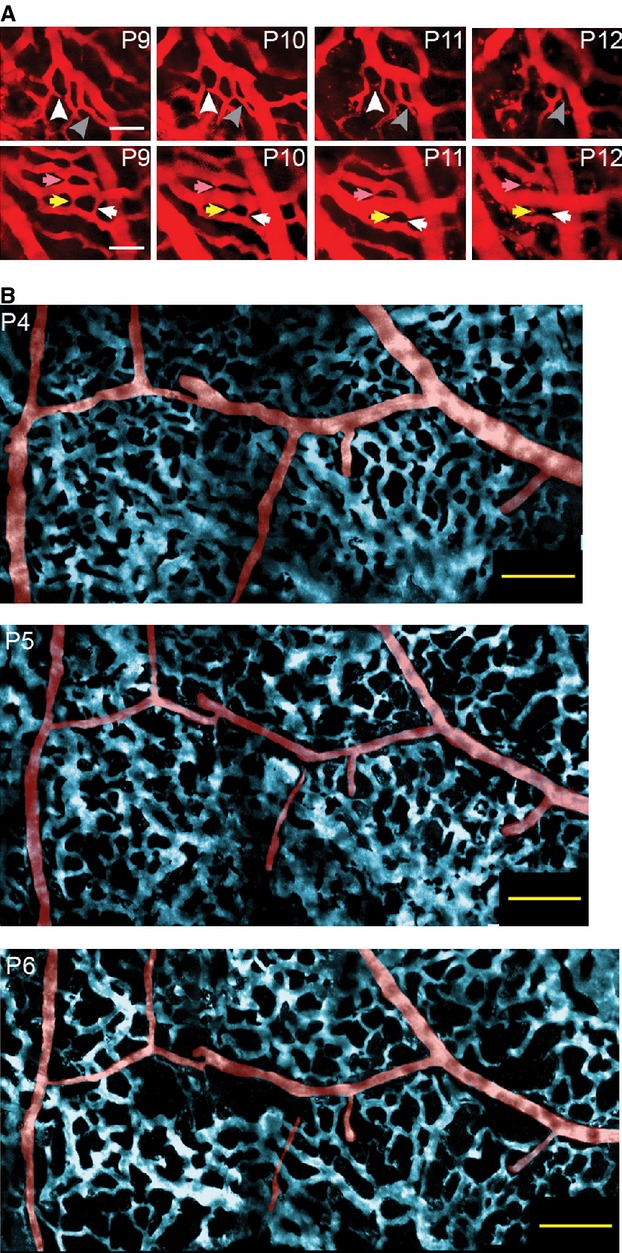
Large chronic windows enable longitudinal imaging of the neonatal pial vasculature. (A) Longitudinal series of images taken from two sites over 4 days showing the gradual pruning of vessels in the venous plexus. Arrows indicate vessels that were pruned. Scale bars 50 *μ*m. (B) False color‐image of pial vasculature, with arteries in red and the venous plexus colored in blue, taken over three consecutive days. There were changes in both the venous plexus, and in the arteriole network. Scale bar 100 *μ*m.

Because we could longitudinally image the same areas in the cortex over several days, we could clearly observe pial vessel pruning, which previously has only been inferred with acute measurements across animals (Wang et al. 1992). Examples of the observed pruning are shown in Figure 2. We observed pruning of both arteries and veins. An example of venous remodeling in two different locations over several days is shown in Figure 2A. Several vessels were removed over the 4 days of imaging, and many more change diameter. An example of arterial pruning in the first postnatal week is shown in Figure 2B. Although portions of the artery constrict enough to no longer be visible under the two‐photon, the visibility of the downstream segment indicated that fluorescently labeled plasma was still perfusing the vessel.

### Relationship between blood flow and vessel remodeling

**Figure 3.**
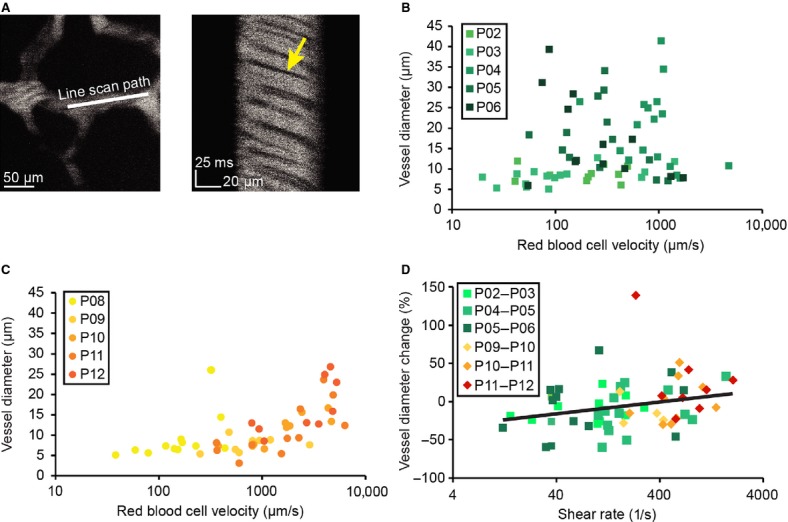
Measures of RBC velocity through individual vessels in awake mice and their relationship to subsequent diameter changes. (A) Measuring red blood cell velocity though an individual vessel. Left, image of the vessel. Line shows position of linescan. Right, space‐time plot of linescan taken at 2 kHz. Red blood cells appear as dark streaks. The velocity of flow determines the angle of the dark streaks. (B) Relationship between RBC velocity and vessel diameters between P2 and P6. (C) Relationship between RBC velocity and vessel diameters between P8 and P12. (D) Relationship between shear rate and subsequent vessel diameter change. The black line is the fit of the relationship between shear rate and % diameter change (%∆D/D), excluding the point having >100% diameter change, and is given by the equation:∆D/D = 15.4*log_10_(shear rate)‐40.9.

We then investigated the relationship between mechanical forces generated by flowing blood and diameter changes in vessels. Early in development, early vascular growth and pruning are thought to respond to shear forces generated by flowing blood (Culver and Dickinson 2010) and to pathological decreases in blood flow (Gruionu et al. 2005, 2012). It has been proposed that hemodynamic forces play a similar role in postnatal development (Wang et al. 1992), but to our knowledge, this has never been tested in a mammalian model at the level of individual vessels during the period of postnatal growth. The forces that a blood vessel experience will be related to the velocity of blood flow in the vessel (Pries et al. 1994). To test the hypothesis that the mechanical forces generated by blood flow determine subsequent diameter changes, we measured centerline red blood cell velocity along the centerline of individual pial venules in awake mice, and looked at subsequent change in diameter 1 day later to determine if the centerline RBC velocity and diameter change were related. We could not directly measure the forces, specifically the shear stress, that blood flow exerts on the vessel walls, as we do not know the viscosity of the blood flowing through the vessel, which can vary with hematocrit (Pries et al. 1994). Instead, we calculated the shear rate from the RBC velocities, which were measured from linescans along the vessel axis using the Radon transform method (Drew et al. 2010a, 2011; Fig. 3A). The shear rate will be proportional to the shear stress. At earlier postnatal ages (P3–6), the velocity of RBCs through pial venules was largely uncorrelated with the diameter of the venules (Fig. 3B). As the animal ages, we observed a closer correspondence between the diameter of the venules and the flow velocity within them (Fig. 3C). We then asked if these changes in vessel diameter were related to the hemodynamic forces that the vessel experienced. In Figure 3D, we have plotted the estimated shear rate (taken to be 4*centerline velocity/vessel diameter, assuming Poiseuille flow; Wang et al. 1992) versus the percentage change in the diameter on the following day. We performed a linear regression on the log‐transformed shear rate, as the shear rate spanned several orders of magnitude. Excluding one outlier, with a diameter change of >100%, there was a significant and positive relationship between shear rate and diameter change (*R*^2^ = 0.09; *P* = 0.022). There was still a positive correlation when the outlier was included, but the correlation was not as strong (*R*^2^ = 0.06, *P* = 0.055). The observed changes in venous diameters were unlikely to be caused by normal physiological fluctuation in venous diameters, as previous measurements in awake mice have shown that venous diameter changes in the absence of prolonged sensory stimulation is negligible (Drew et al. 2011). This weak positive correlation between the diameter change of a vessel and its shear rate suggests that mechanical forces play a role in determining the change of diameter of vessels in the venous plexus.

## Discussion

We have described a method for longitudinally imaging the cerebral vasculature over large areas in neonatal mice using 2PLSM. Using this technique, we observed the pruning of the cerebral vasculature, measured changes in flow dynamics over time, and showed that the shear rate a vessel experiences, as approximated by the measured RBC velocity, was weakly correlated with the vessel's subsequent diameter change. While we saw a relationship between the shear rate a vessels experiences and its subsequent diameter change, this relationship was not strong. There could be several, nonexclusive reasons for the small amplitude of the correlation between the observed diameter change and shear rate. It could be that the time scale over which the vessel integrates mechanical forces was of a longer or shorter scale than the 1 day period over which measurements were made. Other signals, such as transmural pressure (Bakker et al. 2003) and metabolic signals (Secomb and Pries 2011), were likely playing a role as well. Further research will reveal how these signals interact with those generated hemodynamic forces in assembling the vasculature. Future experiments that manipulate the hematocrit in order to increase or decrease blood viscosity (Lucitti et al. 2007) should help elucidate the role of hemodynamic forces in shaping the cerebral vasculature. However, manipulating hematocrit will also affect other variables relevant to vascular development, such as tissue oxygenation (Parpaleix et al. 2013) and nitric oxide levels (Tsoukias and Popel 2002, 2003). Both nitric oxide levels (Whiteus et al. 2014) and oxygenation (Harb et al. 2013) can affect vessel growth, so careful controls will be necessary to correctly interpret the results of such studies.

We think that it is unlikely that the observed changes in venous diameters reflected normal physiological fluctuations. The changes we observed in the diameters of venules were typically >20%, with some vessel showing changes larger than 50% (Fig. 3D). Previous studies in awake mice have found that prolonged sensory stimulation (Drew et al. 2011) and extended bouts of locomotion (Huo et al. in press) both drive venous diameter increases of up to 5–10%. Spontaneous fluctuations in venous diameters in the absence of sensory stimulation or behavior are much smaller (Drew et al. 2011). As the changes in venule diameters we observed during longitudinal imaging of neonates were substantially larger than dilations associated with normal physiological fluctuations, and since they were observed in the absence of locomotion or overt sensory stimulation, it is likely that remodeling of vessels accounted for the changes in venule diameters.

One possible criticism is that the observed disappearance of vessels was not due to pruning, but was caused by the exclusion of dye by a large number of stalled red blood cells. We think the idea that the observed pruning could be attributed to red blood cell blockage is highly unlikely. First, during the rare, and brief occasions where we have observed red blood cells stop in the capillaries of adult mice, the red blood cells do not pack so tightly together that the plasma, which is fluorescently labeled, was completely excluded. This means that the “outlines” of the red blood cells would be clearly visible if the red blood cells did stop in the venules. A blockage by red blood cells would also appear as a decrease in the diameter of a vessel by 100%, which would not account for the diameter changes in venules that we observed (Fig. 3D). While some venules do eventually disappear, the process takes several days (Fig. 2), during which the lumen gradually shrinks. During this time, plasma skimming (Pries et al. 1996) will likely prevent any red blood cells from entering the shrinking venule. Blockage by red blood cells was also not likely to account for reduced diameters of arterioles, as the blood pressure is substantially higher in the arterioles than in the venules, and as the diameters of the arterioles and venules are similar, if there was blockage of red blood cells in the arterioles we would have observed blockage in the venules as well. As we did not observe any prolonged stoppage of red blood cell flow in the venules, it is unlikely that the reduced vessel lumen diameters of arterioles could be attributed to blockage of flow. The nature of the vessel remodeling could be further explored by making use of mice expressing fluorescent proteins in endothelial cells (Harb et al. 2013; Whiteus et al. 2014), allowing visualization of the vessel wall, not just the labeled plasma.

Our window technique should be of use not only in studying blood flow and vascular remodeling during normal growth processes, but also pathological phenomenon like pediatric stroke (Lynch et al. 2002). When combined with the newest generation of genetically encoded calcium indicators (Chen et al., 2013), this chronic, thinned‐skull window in could be used to study the cortical control of sensory‐motor development (Adelsberger et al. 2005), and the development of neurovascular coupling (Colonnese et al. 2007).

## Conflict of Interests

None.
